# Clinical outcomes of children with COVID-19 and appendicitis: a propensity score matched analysis

**DOI:** 10.1007/s00383-024-05817-7

**Published:** 2024-10-08

**Authors:** Marjorie N. Odegard, Olivia A. Keane, Shadassa A. Ourshalimian, Christopher J. Russell, William G. Lee, Makayla L. O’Guinn, Laura M. C. Houshmand, Lorraine I. Kelley-Quon

**Affiliations:** 1 Division of Pediatric Surgery, Department of Surgery, Keck School of Medicine, Children’s Hospital Los Angeles, University of Southern California, 4650 Sunset Blvd, Mailstop #100, Los AngelesLos Angeles, CACA 90027 USA; 2 Division of Pediatric Hospital Medicine, Stanford University School of Medicine, Palo Alto, CA USA; 3 Division of Pediatric Surgery, Cedars Sinai Medical Center, Los Angeles, CA USA; 4 Department of Population and Public Health Sciences, Keck School of Medicine, University of Southern California, Los Angeles, CA USA

**Keywords:** Appendicitis, COVID-19, Outcomes

## Abstract

**Objective:**

Early in the COVID-19 pandemic, many children with appendicitis and COVID-19 were initially treated non-operatively and later underwent interval appendectomy. Currently, children with both appendicitis and COVID-19 frequently undergo upfront appendectomy. The impact of this return to upfront surgical management on patient outcomes is unknown. This study compared outcomes of pediatric patients with and without COVID-19 infection undergoing appendectomy.

**Study design:**

A retrospective cohort study of children < 21y who underwent appendectomy from 3/19/2020 to 7/31/2022 at 50 Pediatric Health Information System children’s hospitals was conducted. Children with documented COVID-19 were identified. Exclusions included preoperative ventilator or supplemental oxygen dependence, and missing data. To evaluate COVID-19 positive versus COVID-19 negative patients, we used a propensity score matched on sociodemographics, comorbidities, laparoscopy, perforation, and hospital. Chi-square and Mann–Whitney *U* tests identified differences between groups in length of stay, postoperative drain placement, 30-day re-admission, and mechanical ventilation requirements.

**Results:**

Overall, 51,861 children of median age 11y (IQR: 8–14) underwent appendectomy, of whom 1,440 (2.3%) had COVID-19. Most were male (60.3%), White (72.1%) and non-Hispanic (61.4%). Public insurance was the most common (47.5%). We created a matched cohort of 1,360 COVID-19 positive and 1,360 COVID-19 negative children. Children with COVID-19 had shorter hospitalizations (1d, IQR: 1–4 vs. 2d, IQR: 1–5, *p* = 0.03), less postoperative peritoneal drain placement (2.4% vs. 4.1%, *p* = 0.01), and fewer 30-day readmissions (9.0% vs. 11.4%, *p* = 0.04). However, no difference in incidence or duration of mechanical ventilation (*p* > 0.05) was detected.

**Conclusions:**

Our findings suggest that upfront appendectomy for children with appendicitis and COVID-19 has similar outcomes compared to children without COVID-19.

**Level of evidence:**

Level III.

**Supplementary Information:**

The online version contains supplementary material available at 10.1007/s00383-024-05817-7.

## Introduction

Early in the coronavirus of 2019 (COVID-19) pandemic, many children in the United States with appendicitis and COVID-19 infection underwent initial nonoperative management [1] (though this was not necessarily the case outside of the United States [2]). Delays in operative management were due to limited operating room resources and staff availability [3] as well as concern for infection of the operative team members due to the aerosols produced during laparoscopy [4]. Additionally, there was concern for potential perioperative morbidity associated with COVID-19 infection as demonstrated in adults [5–8].

Previous studies among children with COVID-19 who underwent surgery demonstrated favorable postoperative results compared to their adult counterparts [9]. A multicenter retrospective study found that children presenting with appendicitis were more likely to experience nonoperative management of appendicitis during the COVID-19 pandemic [1], and several studies found an increase in presentation with perforation [10–12]. Currently, children with both appendicitis and COVID-19 frequently undergo upfront appendectomy and COVID testing only occurs a child appears symptomatic. There have been many studies evaluating pediatric outcomes for appendicitis during the COVID-19 pandemic compared to before the pandemic [1, 3, 10–13]. However, there is a dearth of published studies that directly compare the outcomes of children with COVID-19 who underwent upfront appendectomy compared to children without COVID-19 who underwent upfront appendectomy.

In this propensity score matched retrospective cohort study, we compared children who underwent appendectomy with and without COVID-19 using the Pediatric Health Information System (PHIS) database. Our hypothesis was that children with COVID-19 who undergo appendectomy experience similar clinical outcomes compared to those without COVID-19.

## Methods

### Study design and data collection

We performed a retrospective cohort study that included 50 hospitals (48 after propensity score matching) using data from the PHIS database, an administrative and billing database maintained by Children’s Hospital Association (Lenesa, Kansas and Washington, D.C). The PHIS database contains deidentified clinical and resource utilization data for inpatient and outpatient encounters at United States children’s hospitals. ﻿Data quality is monitored by the Children’s Hospital Association which issues quarterly reports, chart audits, and feedback to participating hospitals. Institutional review board approval was obtained from Children’s Hospital Los Angeles.

**Fig. 1:**
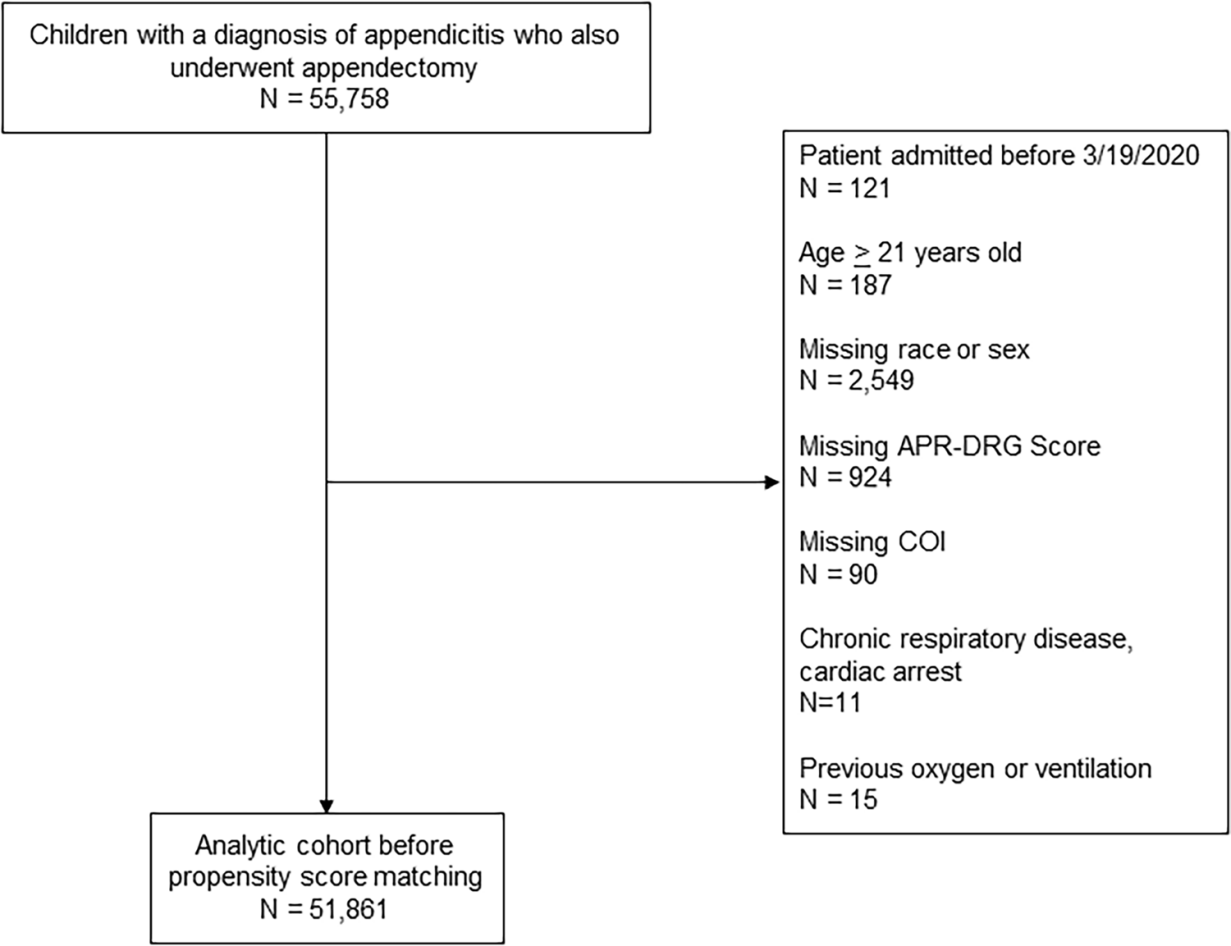
Patient selection flowchart

Our study cohort included children aged 21 years or younger who had both a diagnosis of appendicitis and underwent appendectomy, admitted between 3/19/2020 and 7/31/2022. Our start date was chosen based on when the first state issued a stay-at-home order. Children with pre-hospital ventilation needs, supplemental oxygen needs, or who expired were excluded from the study (Fig. 1). Additionally, children with missing Child opportunity (COI), all patients refined diagnosis-related groups (APR-DRG) illness severity score, sex, or race data were also excluded. Children with chronic respiratory disease and who had cardiac arrest were excluded as there were no children with COVID-19 with whom to match them.

### Outcome measures

Our primary outcome measure was hospital length of stay. Our secondary outcomes included incidence of ventilation and ventilation days, 30-day re-admission, 30-day mortality, and intra-peritoneal drain placement identified by current procedural terminology (CPT) codes 44,900 and 44,901.

### Covariates

Demographic characteristics included age at admission, patient admission type, sex, race, ethnicity, insurance type, hospital region, Child opportunity index (COI), presence of a complex chronic condition [14], and APR-DRG illness severity score. Patient admission type included inpatient, emergency department visit, ambulatory surgery, or observation unit. Race categories included Asian, Black, Other, and White. “Other” race was defined as Native Hawaiian/Pacific Islander, American Indian, and Alaska Native. Insurance type was categorized as private (commercial plans, TRICARE, military insurance), public (Medicare, Medicaid, Children’s Health Insurance Program), and other (any other government insurance, self-pay, charity care, any insurance not previously mentioned).

Children with COVID-19 infection during hospitalization were identified using the International Classification of Diseases, Tenth Revision (ICD-10) codes U07.1 and U07.2. Data extraction from the electronic medical record via chart review was performed on the 555 patients at our institution to assess PHIS’s COVID ICD-10 coding accuracy. We found that PHIS ICD-10 coding reported COVID with 98% accuracy, 100% positive predictive value, 97% negative predictive value, 54% sensitivity, and 100% specificity. These numbers are similar or better than previously published studies [15, 16]. A total of 28 children who tested positive for COVID-19 prior to surgery, and we identified 13 patients who had preoperative COVID diagnoses that PHIS did not identify. Of those 13 patients, 8 did not have COVID ICD codes attached to their encounter, and 1 patient was likely a false-positive test as they tested negative 24 h later.

Diagnosis of appendicitis was identified using ICD-10 codes K35, K35.3, K35.30, K35.31, K35.8, K35.80, K35.89, K35.890, K35.891, K36, and K37. Perforated appendicitis was identified by ICD-10 codes K35.2, K35.20, K35.21, K35.32, and K35.33 [17]. Appendectomy was identified using ICD-10 procedure codes 0DTJ4ZZ and 0DTJ8ZZ for laparoscopic surgery, and 0DTJ0ZZ and 0DTJ7ZZ for open surgery.

Pre-hospital ventilation needs were defined by ICD-10 code Z99.11 and supplemental oxygen needs were defined by ICD-10 code Z99.8. Children with chronic respiratory disease were identified using ICD-10 codes G47.35, I27.82, I43, J84.112, J96.20, and Z90.2. Children who had cardiac arrest were identified using ICD-10 codes I97.121 5A12012, 5A1221Z, and 5A2204Z.

The Child opportunity index (COI) 2.0 is a composite index of 29 indicators of neighborhood resources important to healthy childhood development across three domains: education, health and environment, and social and economic [18]. For this study, COI was measured at the zip code level. Each zip code is assigned a nationally normed neighborhood opportunity score from 1 (lowest) to 100 (highest), and is further categorized into COI quintiles (very low, low, moderate, high, and very high opportunity). The COI is administered by diveresitydatakids.org at the Institute for Child, Youth and Family Policy at the Heller School for Social Policy and Management at Brandeis University (Waltham, MA). [19]

The APR-DRG illness severity score was developed by 3 M Health Information Systems as a measure of illness severity based on clinical similarities and use of hospital resources to compare outcomes across hospitals [20]. Severity classifications include minor, moderate, major, and no class specified.

### Statistical analysis

Categorical variables were described using frequencies and percentages, and continuous variables were described median and interquartile range. Chi-square tests for categorical variables and Mann–Whitney *U* tests for continuous variables were used to compare differences between groups. Given the significant difference in sample size and observed covariates between COVID negative and COVID positive patients, we used propensity score matching to select similar patients for comparison postoperative outcomes. Greedy (1:1 ratio) propensity score matching was performed to match the study group. Patients were matched on age, sex, race, ethnicity, patient admission type, insurance type, Child Opportunity Index, surgical approach, perforation status, hospital, all patients refined diagnosis-related groups (APR-DRG) illness severity, and presence of a complex chronic condition. We felt it necessary to match on race and ethnicity, insurance type, and COI given previous studies demonstrating that children from racial and ethnic minorities [21–25], children with low socioeconomic status [26], and children with public insurance [22, 25] are at higher risk for perforated appendicitis, and therefore at higher risk for complications [27]. All statistical significance tests were conducted with a two-sided α = 0.05. Matching and data analysis was done using SAS software (SAS Institute, Inc, Cary, North Carolina).

## Results

### Unmatched cohort

Before propensity score matching, of the 51,861 children with appendicitis who underwent appendectomy, 1440 (2.3%) were COVID-19 positive. Median age was 11 years old (IQR: 8–14) and the majority of the cohort was male (60.3%). Our cohort was comprised of 1410 (2.7%) Asian children, 3496 (6.7%) Black children, 6,573 children identified as “Other” race, and 37,382 (72.1%) White children. Most identified as non-Hispanic ethnicity (*N* = 31,846, 61.4%). Similar percentages of children had private (N = 23,833, 46%) and public insurance (*N* = 24,629, 46.5%). The United States geographic region that had the most children in our cohort was the South (*N* = 22,117, 42.7%). The COI quintile with the most children was “Very High” (*N* = 11,582, 22.3%). Few children had complex chronic conditions (*N* = 2745, 5.3%), and most children had an APR-DRG illness severity of Minor (*N* = 42,542, 82%). (Table 1).

**Table 1:** Unmatched cohort demographics and outcomes

	Total (*N* = 51,861)		COVID-19 diagnosis				*P* value
			Negative (*N* = 50,421)		Positive (*N* = 1440)		
	*N*	%	*N*	%	*N*	%	
Age at admission, years (median, IQR^a^)	11	8.0–14.0	11	8.0–14.0	11	8.0–14.0	0.50
Length of stay, days (median, IQR)	1	1.0–3.0	1	1.0–3.0	1	1.0–4.0	< 0.01
Patient type							
Inpatient	21,484	41.4	20,818	41.3	666	46.3	< 0.01
Emergency department visit	1,512	2.9	1459	2.9	53	3.7	
Ambulatory surgery	5898	11.4	5792	11.5	106	7.4	
Observation unit	22,967	44.3	22,352	44.3	615	42.7	
Sex							
Male	31,252	60.3	30,393	60.3	859	59.7	0.63
Female	20,609	39.7	20,028	39.7	581	40.4	
Race							
Asian	1,410	2.7	1385	2.8	25	1.7	0.11
Black	3,496	6.7	3402	6.8	94	6.5	
Other^b^	9,573	18.5	9294	18.4	279	19.4	
White	37,382	72.1	36,340	72.1	1042	72.4	
Ethnicity							
Hispanic	18,872	36.4	18,206	36.1	666	46.3	< 0.01
Non-hispanic	31,846	61.4	31,081	61.6	765	53.1	
Unknown	1143	2.2	1134	2.3	< 10	< 0.7	
Insurance							
Private	23,833	46.0	23,254	46.1	579	40.2	< 0.01
Public	24,629	47.5	23,905	47.4	724	50.3	
Other	3399	6.6	262	6.5	137	9.5	
Region							
Midwest	11,359	21.9	11,091	22.0	268	18.6	< 0.01
Northeast	4933	9.5	4858	9.6	75	5.2	
South	22,117	42.7	21,336	42.3	781	54.2	
West	13,452	25.9	13,136	26.1	316	21.9	
Child opportunity index							
Very low	10,686	20.6	10,347	20.5	339	23.5	< 0.01
Low	10,150	19.6	9844	19.5	306	21.3	
Moderate	9899	19.1	9617	19.1	282	19.6	
High	9544	18.4	9296	18.4	248	17.2	
Very high	11,582	22.3	11,317	22.5	265	18.4	
Complex chronic condition	2745	5.3	2666	5.3	79	5.5	0.74
Perforated appendicitis	17,579	33.9	17,021	33.8	558	38.8	< 0.01
Open surgical approach	937	1.8	906	1.8	31	2.2	0.32
APR-DRG Illness Severity							
No class specified	46	0.1	46	0.1	0	0.0	< 0.01
Minor	42,542	82.0	42,522	84.3	20	1.4	
Moderate	7773	15.0	6606	13.1	1,167	81.0	
Major	1198	2.3	976	1.9	222	15.4	
Extreme	302	0.6	271	0.5	31	2.2	
Drain placement	702	1.4	669	1.3	33	2.3	< 0.01
Mechanically ventilated (Y/N)	132	0.3	121	0.2	11	0.8	< 0.01
Duration of mechanical ventilation (median, IQR)	3	1.0–6.0	3	1.0–6.0	5	3.0–7.0	0.26
Readmission within 30 days	4038	7.8	3907	7.8	131	9.1	0.06
Mortality	< 10	< 0.02	< 10	< 0.02	< 10	< 0.7	0.16

^a^IQR = interquartile range

^b^Native Hawaiian/Pacific Islander, American Indian, and Alaska Native

On bivariate analysis COVID-19 positive children who underwent appendectomy notably had more Hispanic patients (*N* = 666, 46.3% vs *N* = 18,206, 36.1%, *p* < 0.01) and more children with public insurance (*N* = 724, 50.3%, vs *N* = 23,905, 47.4%, *p* < 0.01) compared to children without COVID-19. Children with COVID-19 also were most often from the South (*N *= 781, 54.2%), most often resided in a Very Low COI neighborhood (*N* = 339, 23.5%), and the majority had a Moderate APR-DRG illness severity score (*N* = 1,167, 81%). Finally, children with COVID-19 had higher rates of perforated appendicitis (*N* = 558, 38.8%, vs *N* = 17,021, 33.8%, *p* < 0.01), postoperative drain placement (*N* = 33, 2.3%, vs *N* = 669, 1.3%, *p* < 0.01), and incidence of mechanical ventilation (*N* = 11, 0.8%, vs *N* = 121, 0.2%, *p* < 0.01) (Table 1).

### Propensity score matched cohort

A total of 2720 patients were included in the propensity score matched analysis. On bivariate analysis of the matched cohort, there was no significant difference between COVID-19 negative and COVID-19 positive groups with respect to demographics, comorbidities, and clinical/surgical variables used for matching (all *p* > 0.05). Children with COVID-19 who underwent appendectomy had significantly shorter hospital length of stay (1 day, IQR: 1–4 vs. 2 days, IQR: 1–5, *p* = 0.03), but no difference in incidence (0.8% vs. 0.5%, *p* = 0.34) or duration (5 days, IQR: 3–7 vs. 2 days, IQR: 1–5, *p* = 0.31) of mechanical ventilation. Children with COVID-19 who underwent appendectomy were less likely to require postoperative peritoneal drain placement (*N* = 33, 2.4% vs. *N* = 56, 4.1%, *p* = 0.01), and had fewer 30-day readmissions (*N* = 123, 9.0% vs. *N* = 155, 11.4%, *p* = 0.04). There was no significant difference in mortality (*N* < 10, < 0.7% vs *N* = 0, 0%, *p* > 0.99) (Table 2).

**Table 2:** Matched cohort demographics and outcomes

	COVID-19 diagnosis				*P* value
	Negative (*N* = 1,360)		Positive (*N* = 1,360)		
	*N*	%	*N*	%	
Age at admission, years (median, IQR^a^)	11	8.0–14.0	11	8.0–14.0	0.95
Length of stay, days (median, IQR)	2	1.0–5.0	1	1.0–4.0	0.02
Patient type					
Inpatient	665	48.9	666	49.0	0.98
Emergency department visit	54	4.0	53	3.9	
Ambulatory surgery	110	8.1	105	7.7	
Observation unit	531	39.0	536	39.4	
Sex					
Male	810	59.6	817	60.1	0.78
Female	550	40.4	543	39.9	
Race					
Asian	21	1.5	24	1.8	0.95
Black	93	6.8	94	6.9	
Other^b^	280	20.6	272	20.0	
White	966	71.0	970	71.3	
Ethnicity					
Hispanic	582	42.8	598	44.0	0.59
Non-hispanic	765	56.3	753	55.4	
Unknown	13	1.0	< 10	< 0.7	
Insurance					
Private	558	41.0	559	41.1	1.00
Public	690	50.7	688	50.6	
Other	112	8.2	113	8.3	
Region					
Midwest	279	20.5	265	19.5	0.76
Northeast	84	6.2	75	5.5	
South	702	51.6	714	52.5	
West	295	21.7	306	22.5	
Child opportunity index					
Very low	305	22.4	309	22.7	0.96
Low	292	21.5	283	20.8	
Moderate	273	20.1	272	20.0	
High	227	16.7	240	17.7	
Very high	263	19.3	256	18.8	
Complex chronic condition	85	6.3	79	5.8	0.63
Perforated appendicitis	559	41.1	557	41.0	0.94
Open surgical approach	29	2.1	31	2.3	0.79
APR-DRG illness severity					
No class specified	0	0	0	0	0.55
Minor	21	1.5	20	1.5	
Moderate	1081	79.5	1097	80.7	
Major	234	17.2	212	15.6	
Extreme	24	1.8	31	2.3	
Drain placement	56	4.1	33	2.4	0.01
Mechanically ventilated (Y/N)	< 10	< 0.7	11	0.8	0.34
Duration of mechanical ventilation (days) (median, IQR)	2	1.0–5.0	5	3.0–7.0	0.31
Readmission within 30 days	155	11.4	123	9.0	0.04
Mortality	0	0	< 10	< 0.7%	> 0.99

^a^IQR = interquartile range

^b^Native Hawaiian/Pacific Islander, American Indian, and Alaska Native

## Discussion

After propensity score matching, we found that compared to children without COVID-19 who underwent appendectomy, children with COVID-19 who underwent appendectomy had no difference in outcomes. However, children with COVID-19 who underwent appendectomy had shorter hospitalizations, less postoperative peritoneal drain placement, and fewer 30-day readmissions. Our findings demonstrate that upfront appendectomy is still a safe option for children who are positive for COVID-19.

There are several explanations for the observed outcomes for the COVID-19 positive children could be decreased healthcare utilization that has been observed during the COVID-19 pandemic. In a systematic review, Moynihan et al. found that healthcare utilization had decreased by one-third globally, since the COVID-19 pandemic had started [28]. Meanwhile, Antoon et al. found that encounters for children with respiratory disease decreased during the COVID-19 pandemic [29]. Fritz et al. found that acute care utilization for children decreased during the COVID-19 pandemic including those with gastrointestinal surgery related diagnoses, and children from the lowest COI quintiles had the highest decrease in healthcare utilization levels [30]. Notably, in our unmatched cohort comparisons, the COVID-19 positive children had a higher proportion of patients with lower COI levels. While these studies’ findings may partially explain the shorter hospital stay and decreased 30-day re-admission rate among COVID-19 positive children in our matched cohort, they do not necessarily account for the decreased incidence of intra-peritoneal drain placement. Another explanation of our outcomes could be confounding by indication bias: The sickest COVID-19 patients who were symptomatic may have been managed non-operatively due to perceived increased risk of perioperative morbidity and mortality and therefore not included in our study cohort.

The decrease in intra-peritoneal drain placement could again be due to excluding patents who were managed non-operatively from our cohort, though Hedge et al. similarly demonstrated that drain placements decreased during COVID-19 even among pediatric appendicitis patients who were managed non-operatively [1]. Children who present with perforated appendicitis are more likely to develop an intra-abdominal abscess [27] which may require drain placement. Some single-center studies have found higher rates of presentation with perforated appendicitis during the COVID-19 pandemic, [10–12] while other single-center [13] and statewide studies [31] have found no increase in presentation with perforated appendicitis. Our unmatched cohort comparison found increased rates of perforated appendicitis and a higher incidence of drain placement in children with COVID-19. After propensity score matching, accounting for perforation status, the incidence of drain placement was observed to decrease in the COVID-19 positive cohort, suggesting that COVID-19 status alone is not associated with developing postoperative infection needing intra-peritoneal drain placement.

Our study has several important limitations to consider when interpreting our results. Our data are from 50 standalone children’s hospitals across the United States which may limit generalizability to other settings. Also, the retrospective nature of the PHIS database also puts our study at risk of potential misclassification bias. Due to PHIS being an administrative and billing database, we are unable to distinguish whether patients who had COVID-19 were symptomatic and unable to confirm the diagnoses of appendicitis with operative or pathology reports. We did try to mitigate misdiagnosis of appendicitis by only including children who had both a diagnosis code for appendicitis and a procedure code for appendectomy. PHIS includes patients up to 21 years old and thus was the age range used for our study, which may have influenced the results reported. However, we feel that this impact of including patients 18–21 years is minimal as the median age of in our study was 11 (IQR 8–14 years), with no statistically significant difference between cohorts before or after propensity score matching. Our institutional chart review showed that PHIS had a low sensitivity when reporting COVID-19 ICD-10 codes, though most of these diagnoses codes were not picked up, because the patient did not have a COVID-19 ICD code associated with their encounter. Therefore, there is a risk that the total number of COVID-19 positive patients was underreported. Our study time period took place over the rollout of vaccine eligibility for different age groups of children, though we did try to account for this by including age as part of our matching criteria. We do acknowledge that the same APR-DRG severity score may have a different clinical implication across different illness types which could limit the utility of matching on APR-DRG score. However as all children in our cohort had appendicitis, we also matched on perforation status to mitigate the clinical differences in APR-DRG scores. On bivariate analysis of our matched cohort, there was no statistically significant difference in APR-DRG severity score distribution by COVID-19 status among children with perforated appendicitis (*p* = 0.54), or children with non-perforated appendicitis (*p* = 0.09) (Supplemental Table 1). Finally, the difference in days of mechanical ventilation in the COVID-19 positive children (5 days) was not statistically significant compared to the COVID-19 negative children (2 days). However, we do acknowledge that additional 3 days of mechanical ventilation are clinically significant and we may not have found statistical significance due to low prevalence of this outcome.

## Conclusions

In this propensity score matched analysis, we found that upfront appendectomy for children with appendicitis and COVID-19 was not associated with worse clinical outcomes. Children with COVID-19 who underwent appendectomy had shorter hospital length of stay, lower need for postoperative drain placement, and lower 30-day readmissions. These findings could partially be explained by decreased healthcare utilization during the COVID-19 pandemic.

## Supplementary Information

Below is the link to the electronic supplementary material.

Supplementary file1 (DOCX 14 KB)

## Data Availability

The dataset generated for this study is not available for public use.

## Abbreviations

COVID-19: Coronavirus of 2019
PHIS: Pediatric health information system
COI: Child opportunity
APR-DRG: All patients refined diagnosis-related groups

## References

[1] Hegde B, Garcia E, Hu A et al (2022) Management of pediatric appendicitis during the COVID-19 pandemic: a nationwide multicenter cohort study. J Pediatr Surg. 10.1016/J.JPEDSURG.2022.08.005 36075771 10.1016/j.jpedsurg.2022.08.005 PMC9374489

[2] Van Amstel P, El Ghazzaoui A, Hall NJ et al (2022) Paediatric appendicitis: international study of management in the COVID-19 pandemic. Br J Surg 109(11):1044. 10.1093/BJS/ZNAC239 36240511 10.1093/bjs/znac239 PMC9384519

[3] Kvasnovsky CL, Shi Y, Rich BS et al (2021) Limiting hospital resources for acute appendicitis in children: lessons learned from the U.S. epicenter of the COVID-19 pandemic. J Pediatr Surg. 10.1016/J.JPEDSURG.2020.06.024 32620267 10.1016/j.jpedsurg.2020.06.024 PMC7309720

[4] Tummers FHMP, Draaisma WA, Demirkiran A et al (2022) Potential risk and safety measures in laparoscopy in COVID-19 positive patients. Surg Innov 29(1):73. 10.1177/15533506211003527 33788655 10.1177/15533506211003527 PMC8948368

[5] Brown WA, Moore EM, Watters DA et al (2021) Mortality of patients with COVID-19 who undergo an elective or emergency surgical procedure: a systematic review and meta-analysis. ANZ J Surg 91(1–2):33–41. 10.1111/ANS.16500 33369009 10.1111/ans.16500

[6] Rasslan R, dos Santos JP, Menegozzo CAM et al (2021) Outcomes after emergency abdominal surgery in COVID-19 patients at a referral center in Brazil. Updates Surg 73(2):763–768. 10.1007/S13304-021-01007-5/TABLES/4 33625679 10.1007/s13304-021-01007-5 PMC7903871

[7] Osorio J, Madrazo Z, Videla S et al (2021) Analysis of outcomes of emergency general and gastrointestinal surgery during the COVID-19 pandemic. Br J Surg 108(12):1438–1447. 10.1093/BJS/ZNAB299 34535796 10.1093/bjs/znab299

[8] Knisely A, Zhou ZN, Wu J et al (2021) Perioperative Morbidity and Mortality of Patients With COVID-19 Who Undergo Urgent and Emergent Surgical Procedures. Ann Surg 273(1):34. 10.1097/SLA.0000000000004420 33074900 10.1097/SLA.0000000000004420 PMC7737869

[9] Mehl SC, Loera JM, Shah SR et al (2021) Favorable postoperative outcomes for children with COVID-19 infection undergoing surgical intervention: experience at a free-standing children’s hospital. J Pediatr Surg 56(11):2078–2085. 10.1016/J.JPEDSURG.2021.01.033 33581882 10.1016/j.jpedsurg.2021.01.033 PMC7838581

[10] Esparaz JR, Chen MK, Beierle EA et al (2021) Perforated appendicitis during a pandemic: the downstream effect of COVID-19 in children. J Surg Res 268:263–266. 10.1016/J.JSS.2021.07.008 34392179 10.1016/j.jss.2021.07.008 PMC8299184

[11] Li C, Saleh A (2022) Effect of COVID-19 on pediatric appendicitis presentations and complications. J Pediatr Surg 57(5):861–865. 10.1016/J.JPEDSURG.2021.12.047 35093252 10.1016/j.jpedsurg.2021.12.047

[12] Gerall CD, DeFazio JR, Kahan AM et al (2021) Delayed presentation and sub-optimal outcomes of pediatric patients with acute appendicitis during the COVID-19 pandemic. J Pediatr Surg 56(5):905–910. 10.1016/J.JPEDSURG.2020.10.008 33220973 10.1016/j.jpedsurg.2020.10.008 PMC7569380

[13] Nassiri AM, Pruden RD, Holan CA et al (2022) Pediatric appendicitis in the time of the COVID-19 pandemic: a retrospective chart review. J Am Coll Emerg Physicians Open. 10.1002/EMP2.12722 35462960 10.1002/emp2.12722 PMC9019144

[14] Feudtner C, Feinstein JA, Zhong W et al (2014) Pediatric complex chronic conditions classification system version 2: updated for ICD-10 and complex medical technology dependence and transplantation. BMC Pediatr. 10.1186/1471-2431-14-199 25102958 10.1186/1471-2431-14-199 PMC4134331

[15] Lynch KE, Viernes B, Gatsby E et al (2021) Positive predictive value of COVID-19 ICD-10 diagnosis codes across calendar time and clinical setting. Clin Epidemiol 13:1011. 10.2147/CLEP.S335621 34737645 10.2147/CLEP.S335621 PMC8558427

[16] Bhatt AS, McElrath EE, Claggett BL et al (2021) Accuracy of ICD-10 diagnostic codes to identify COVID-19 among hospitalized patients. J Gen Intern Med 36(8):2532. 10.1007/S11606-021-06936-W 34100236 10.1007/s11606-021-06936-w PMC8183587

[17] AHRQ Quality Indicators^TM^ (AHRQ QI^TM^) ICD-10-CM/PCS Specification v2018. Accessed September 16, 2022. www.qualityindicators.ahrq.gov

[18] Noelke C, Mcardle N, Baek M, et al. Child Opportunity Index 2.0. Published online 2020.

[19] Child Opportunity Index (COI) | diversitydatakids.org. Accessed May 27, 2022. https://www.diversitydatakids.org/child-opportunity-index

[20] 3M^TM^ All Patient Refined Diagnosis Related Groups (APR DRGs) | 3M. Accessed May 28, 2023. https://www.3m.com/3M/en_US/health-information-systems-us/drive-value-based-care/patient-classification-methodologies/apr-drgs/

[21] Kelley-Quon LI, Tseng CH, Jen HC et al (2013) Hospital type as a metric for racial disparities in pediatric appendicitis. J Am Coll Surg 216(1):74–82. 10.1016/J.JAMCOLLSURG.2012.09.018 23177269 10.1016/j.jamcollsurg.2012.09.018

[22] Ponsky T, Huang Z, Kittle K et al (2004) Hospital- and patient-level characteristics and the risk of appendiceal rupture and negative appendectomy in children. JAMA 292(16):1977–1982. 10.1001/JAMA.292.16.1977 15507583 10.1001/jama.292.16.1977

[23] Guagliardo M, Teach S, Huang Z et al (2003) Racial and ethnic disparities in pediatric appendicitis rupture rate. Acad Emerg Med 10(11):1218–1227. 10.1111/J.1553-2712.2003.TB00606.X 14597498 10.1111/j.1553-2712.2003.tb00606.x

[24] Jablonski J, Guagliardo M (2005) Pediatric appendicitis rupture rate: a national indicator of disparities in healthcare access. Popul Health Metr. 10.1186/1478-7954-3-4 15871740 10.1186/1478-7954-3-4 PMC1156944

[25] Smink D, Fishman S, Kleinman K et al (2005) Effects of race, insurance status, and hospital volume on perforated appendicitis in children. Pediatrics 115(4):920–925. 10.1542/PEDS.2004-1363 15805365 10.1542/peds.2004-1363

[26] Bratu I, Martens P, Leslie W et al (2008) Pediatric appendicitis rupture rate: disparities despite universal health care. J Pediatr Surg 43(11):1964–1969. 10.1016/J.JPEDSURG.2008.05.013 18970925 10.1016/j.jpedsurg.2008.05.013

[27] Kelly KN, Fleming FJ, Aquina CT et al (2014) Disease severity, not operative approach, drives organ space infection after pediatric appendectomy. Ann Surg 260(3):466–473. 10.1097/SLA.0000000000000874 25115422 10.1097/SLA.0000000000000874

[28] Moynihan R, Sanders S, Michaleff ZA et al (2021) Impact of COVID-19 pandemic on utilisation of healthcare services: a systematic review. BMJ Open 11(3):e045343. 10.1136/BMJOPEN-2020-045343 33727273 10.1136/bmjopen-2020-045343 PMC7969768

[29] Antoon JW, Williams DJ, Thurm C et al (2021) The COVID-19 pandemic and changes in healthcare utilization for pediatric respiratory and nonrespiratory illnesses in the united states. J Hosp Med 16(5):294. 10.12788/JHM.3608 33734976 10.12788/jhm.3608 PMC8086992

[30] Fritz CQ, Fleegler EW, DeSouza H et al (2022) Child opportunity index and changes in pediatric acute care utilization in the COVID-19 pandemic. Pediatrics. 10.1542/PEDS.2021-053706 35233618 10.1542/peds.2021-053706

[31] Theodorou CM, Beres AL, Nguyen M et al (2021) Statewide impact of the COVID pandemic on pediatric appendicitis in California: a multicenter study. J Surg Res 267:132–142. 10.1016/J.JSS.2021.05.023 34147003 10.1016/j.jss.2021.05.023 PMC8674370

