# Supplementary material for: Clinical outcomes of children with COVID-19 and appendicitis: a propensity score matched analysis
Source: Pediatr Surg Int. 2024 Oct 8;40(1):266. doi: 10.1007/s00383-024-05817-7 (PMC11461633; doi:10.1007/s00383-024-05817-7)
Supplement: Supplementary file 1 — Supplementary file1 (DOCX 14 KB) [file 383_2024_5817_MOESM1_ESM.docx]

**Supplemental Table 1. APR-DRG Illness Severity Distribution in the Matched Cohort by Perforation Status**

| PERFORATED APPENDICITIS | | | |
| --- | --- | --- | --- |
|  | **COVID-19 Diagnosis** | |  |
|  | **Negative (N=559)** | **Positive (N=557)** | **P-value** |
| **APR-DRG Illness Severity** |  |  |  |
| Minor | 21 | 20 |  |
| Moderate | 1081 | 1097 |  |
| Major | 234 | 212 |  |
| Extreme | 24 | 31 |  |
|  |  |  | 0.54 |
| NON-PERFORATED APPENDICITIS | | | |
|  | **COVID-19 Diagnosis** | |  |
|  | **Negative (N=801)** | **Positive (N=803)** | **P-value** |
| **APR-DRG Illness Severity** |  |  |  |
| Minor | 20 | 20 |  |
| Moderate | 695 | 724 |  |
| Major | 81 | 53 |  |
| Extreme | 5 | 6 |  |
|  |  |  | 0.09 |
